# Complete Genome Sequence of Klebsiella pneumoniae Strain ATCC 43816

**DOI:** 10.1128/MRA.01441-20

**Published:** 2021-02-04

**Authors:** James A. Budnick, X. Renee Bina, James E. Bina

**Affiliations:** a University of Pittsburgh School of Medicine, Department of Microbiology and Molecular Genetics, Pittsburgh, Pennsylvania, USA; Loyola University Chicago

## Abstract

Klebsiella pneumoniae is a member of *Enterobacteriaceae* that causes a multitude of infections in compromised and healthy individuals. The rise of hypervirulent and multiple-drug-resistant K. pneumoniae strains has made this organism a global health threat. Here, we report the complete genome sequence of K. pneumoniae strain ATCC 43816.

## ANNOUNCEMENT

Klebsiella pneumoniae is a member of *Enterobacteriaceae* that can cause both community-acquired and nosocomial infections in humans. K. pneumoniae can cause a wide range of life-threatening infections, including pneumonia, bacteremia, urinary tract infection, and liver abscesses (1). K. pneumoniae is generally considered an opportunistic pathogen, but recently, highly virulent strains capable of causing community-acquired infections in healthy individuals have emerged (2). This change in pathogenic potential among some isolates has coincided with the rapid evolution and dissemination of multiple drug resistance in K. pneumoniae. The convergence of antimicrobial resistance and hypervirulence in K. pneumoniae has made this organism a significant global health threat (3). Here, we present the genome sequence of K. pneumoniae ATCC 43816, a strain that is commonly used to study K. pneumoniae pathogenesis. The availability of the ATCC 43816 genome sequence will facilitate future studies on the pathobiology of this organism.

K. pneumoniae strain ATCC 43816 (serotype O1:K2) was obtained from the American Type Culture Collection (www.atcc.org). To sequence the K. pneumoniae ATCC 43816 genome, total genomic DNA was isolated using the MasterPure kit (Epicentre, Madison, WI) according to the manufacturer’s instructions from a saturated culture that was grown overnight at 37°C in lysogeny broth. The resulting genomic DNA was then sent to SNPsaurus (Eugene, OR) for whole-genome sequencing on a PacBio Sequel II system. The genomic DNA was processed according to the PacBio protocol for preparing multiplexed microbial Sequel SMRTbell libraries for the PacBio Sequel system and sequenced with SMRT Link 6.0 sequencing chemistry and a library that was size selected for fragments of ≥7 kb with a 30-hour read time using a fraction of a single-molecule real-time sequencing (SMRT) cell. The sequencing library was prepared using a PacBio SMRTbell express template prep kit 2.0 with genomic DNA that was size selected using the BluePippin system (Sage Sciences, Beverly, MA) according to the manufacturer’s instructions. This resulted in 326,237 sequencing reads with a 7,286-bp mean read length to generate 2.38 × 10^9^ total bases that were *de novo* assembled and polished by Flye 2.8 using the default settings (4). The total assembled length was 5,362,708 bp with a read depth of 443. Flye 2.8 identified one closed circular contig of 5,362,708 bp. The assembly quality was assessed using BUSCO version 3 with the bacteria_odb10 data set (creation date, 3 June 2020) and was 98.4% complete, with 98.4% of the genome single copy and 0% duplicated (5). The 5.4-Mb genome had a GC content of 57.4%. The genome was annotated by the NCBI Prokaryotic Genome Annotation Pipeline version 4.13 (6).

### Data availability.

The K. pneumoniae strain ATCC 43816 genome sequence was deposited in GenBank under BioProject PRJNA675363 with the accession number CP064352. The raw sequencing results were deposited into the NCBI SRA database under the accession number SRR13008124.
